# Alpha-mannosidosis: correlation between phenotype, genotype and mutant MAN2B1 subcellular localisation

**DOI:** 10.1186/s13023-015-0286-x

**Published:** 2015-06-06

**Authors:** Line Borgwardt, Hilde Monica Frostad Riise Stensland, Klaus Juul Olsen, Flemming Wibrand, Helle Bagterp Klenow, Michael Beck, Yasmina Amraoui, Laila Arash, Jens Fogh, Øivind Nilssen, Christine I Dali, Allan Meldgaard Lund

**Affiliations:** Department of Clinical Genetics, Centre for Inherited Metabolic Diseases, Copenhagen University Hospital Rigshospitalet, 9 Blegdamsvej, 2100 Copenhagen, Denmark; Department of Medical Genetics, Division of Child and Adolescent Health, University Hospital of North Norway, Tromsø, Norway; Larix, CRO, Ballerup, Denmark; Zentrum für Kinder - und Jugendmedizin, Villa Metabolica, Mainz, Germany; Zymenex A/S (Chiesi Group), Hilleroed, Denmark; Department of Clinical Medicine, Medical Genetics, University of North Norway, Tromsø, Norway

**Keywords:** Alpha-mannosidosis, *MAN2B1*, Genotype-phenotype correlation, CNS involvement

## Abstract

**Background:**

Alpha-mannosidosis is caused by mutations in *MAN2B1*, leading to loss of lysosomal alpha-mannosidase activity. Symptoms include intellectual disabilities, hearing impairment, motor function disturbances, facial coarsening and musculoskeletal abnormalities.

**Methods:**

To study the genotype-phenotype relationship for alpha-mannosidosis 66 patients were included. Based on the predicted effect of the mutations and the subcellular localisation of mutant MAN2B1 in cultured cells, the patients were divided into three subgroups.

Clinical and biochemical data were collected. Correlation analyses between each of the three subgroups of genotype/subcellular localisation and the clinical and biochemical data were done to investigate the potential relationship between genotype and phenotype in alpha-mannosidosis.

Statistical analyses were performed using the SPSS software. Analyses of covariance were performed to describe the genotype-phenotype correlations. The phenotype parameters were modelled by the mutation group and age as a covariate. P values of <0.05 were considered as statistically significant.

**Results:**

Complete *MAN2B1* genotypes were established for all patients. We found significantly higher scores in the Leiter-R test, lower concentrations of CSF-oligosaccharides, higher point scores in the Bruininks-Oseretsky Test of Motor Proficiency subtests (BOT-2); Upper limb coordination and Balance, and a higher FVC% in patients in subgroup 3, harbouring at least one variant that allows localisation of the mutant MAN2B1 protein to the lysosomes compared to subgrou 2 and/or subgroup 1 with no lysosomal localization of the mutant MAN2B1 protein.

**Conclusion:**

Our results indicate a correlation between the *MAN2B1* genotypes and the cognitive function, upper limb coordination, balance, FVC% and the storage of oligosaccharides in CSF. This correlation depends on the subcellular localisation of the mutant MAN2B1 protein.

## Background

Alpha-mannosidosis is caused by pathogenic sequence variants in *MAN2B1*, leading to loss of lysosomal alpha-mannosidase activity. There is a broad phenotypic variation of manifestations, including intellectual disabilities, hearing impairment, motor function disturbances, facial coarsening, musculoskeletal abnormalities, and immune deficiency [1, 2].

Together with other lysosomal exoglycosidases, alpha-mannosidase cleaves sugar chains in a specific sequence as a part of the glycoprotein degradation. After degradation, the components of the sugar chains are exported from the lysosomes into the cytosol and reutilized [3]. Alpha-mannosidase deficiency causes a block in the degradation of glycoproteins and thereby a progressive lysosomal accumulation of soluble mannose-rich oligosaccharides in all tissues, resulting in impaired cellular function and apoptosis [4].

*MAN2B1* comprises 24 exons and encodes a 1011 amino acid polypeptide [5]. The MAN2B1 polypeptide is post-translationally modified in the endoplasmic reticulum (ER), and during maturation and endosomal transport of MAN2B1 to the lysosomes it is proteolytically cleaved into three major polypeptides named “abc”, “d” and “e” of 70, 42 and 15 kDa, respectively [6]. Further specific, processing of the 70 kDa subunit results in a total of five different polypeptides [7].

Depending on the causative *MAN2B1* mutation, mutant MAN2B1 proteins have been detected in subcellular compartments such as ER and lysosomes. For instance, the protein can be folded incorrectly and arrested in the ER, or it can be folded correctly and transported to the lysosomes in an inactive form [8, 9].

A total of 127 *MAN2B1* disease-associated mutations have been reported (HGMD® Professional 2015.1 [10]. The mutations are scattered throughout the coding region and include missense mutations, nonsense mutations, frame-shifting small insertions/duplications/deletions, in-frame duplications, intronic splice site mutations and large deletions. In a recent study, 96 alpha-mannosidosis-associated mutations were reported in 130 unrelated patients from 30 countries [11]. Most of these mutations were private, but three mutations, c.2248C>T (p.Arg750Trp), c.1830+1G>C and c.2426 T>C (p.Leu809Pro), were relatively frequent, and accounted for approximately 27 %, 5 % and 3 %, respectively, of the disease alleles [11].

At present, there is no clear relationship between genotype and severity of the disease. The phenotypic variability is high, even between siblings with identical genotypes [1, 12–15]. The molecular basis of alpha-mannosidase deficiency and the phenotype has not previously been studied systematically. However, results from two studies [11, 16] indicated that there was no apparent correlation between mutations and clinical phenotypes.

To study the genotype-phenotype correlation for alpha-mannosidosis further, we performed mutation analysis and investigated the potential relationship between the consequences of *MAN2B1* mutations and the results of motor function tests, cognitive test, and biochemical tests, including alpha-mannosidase activity for each of the 66 patients included in the study.

## Materials and methods

Data presented in this paper are based on baseline data from rhLAMAN-01; a natural history study of alpha-mannosidosis and on baseline data from two randomised clinical trials studying the efficacy and safety of enzyme replacement therapy (ERT) with a recombinant human alpha-mannosidase for patients with alpha-mannosidosis (rhLAMAN-02 (EudraCT number: 2010-022084-36) [17, 18]; and rhLAMAN-05 (EudraCT number: 2012-000979-17) (unpublished data).

### Patients

66 patients (57 unrelated) with clinically and enzymatically confirmed alpha-mannosidosis, age 5-42, were included. The majority of the patients were from Europe (60), four originated from North Africa and two from Pakistan. All patients included had the attenuated form of alpha-mannosidosis (type II). 45 patients had previously been included in rhLAMAN-01 [18], and 35 patients in the rhLAMAN-02 or rhLAMAN-05 studies.

The clinical trials were performed in compliance with the principles of the Declaration of Helsinki, ICH GCP guidelines.

### Mutation analysis of the *MAN2B1* gene

Mutation analysis of the *MAN2B1* gene and determination of the subcellular localisation of mutant MAN2B1 protein were performed as described in Riise Stensland *et al.* [11]. Briefly, the 24 *MAN2B1* exons, corresponding exon-intron borders and parts of the 5’- and 3’- untranslated regions were sequenced using the Sanger method. When possible, parents were analysed for the mutations found in their children in order to confirm their carrier status and the allelic phase of the mutations. The software Alamut Visual version 2.5-1 (Interactive Biosoftware) was used to aid in the interpretiation of novel variants. The effect of novel (potential) splice-site mutations was studied on cDNA synthesized from RNA isolated from peripheral blood cells. The effect of novel missense mutations was studied by expression in cultured cells as described in Riise Stensland *et al.* [11] and slightly modified from Kuokkanen *et al.* [6]. Briefly, the mutant *MAN2B1* mutations were constructed by site-directed mutagenesis of the WT *MAN2B1* cDNA inserted into the pcDNA3.1- vector and expressed in COS-7 cells and/or HeLa cells. The mutant MAN2B1 protein variants were assayed for MAN2B1 activity and the subcellular processing was determined by western blot using rabbit anti-recombinant human MAN2B1 antibodies. For subcellular localisation, HeLa-cells were grown in 8-well micro-slide chambers (Ibidi, Germany), fixated in ice-cold methanol, blocked in PBS with 0.5 % BSA and stained using rabbit anti-denatured bovine MAN2B1, mouse anti-LAMP1 and mouse anti-PDI (primary antibodies) and Alexa 488 goat anti-rabbit and Alexa 568 goat anti-mouse as secondary antibodies. A confocal microscope (Zeiss LSM780, Carl Zeiss Microscopy GmbH, Germany) was used to capture Z-stack images of the transfected cells.

Based on the results from the mutation analysis and predicted (null mutations) or determination of subcellular MAN2B1 protein localisation, patients were divided into three subgroups:

I. Two null-mutations (nonsense, frameshift, large truncations). Subcellular localisation of the mutant MAN2B1 proteins was not studied.
II. At least one missense mutation (or in-frame deletion/duplication of 1-5 amino acids) with the MAN2B1 protein localised to the endoplasmic reticulum (ER) (ie: ER/ER, ER/null).
III. At least one missense mutation (or in-frame deletion/duplication of 1-5 amino acids) with the MAN2B1 protein localised to the lysosomes (lyso) (ie: lyso/lyso, lyso/ER, lyso/null).

### Collection of clinical and biochemical data

RhLAMAN-01 data were collected at four European centres. RhLAMAN-02 and rhLAMAN-05 data were collected at one center. Clinical and biochemical data were collected prospectively. Assessments, including six-minutes-walk test (6-MWT), pulmonary function test, alpha-mannosidase enzyme activity and audiometry were made in all patients included in rhLAMAN-01, rhLAMAN-02 and rhLAMAN-05 (n = 66). Assessments, including Three-minutes-stair-climb-test (3-MSCT), The Bruininks-Oseretsky Test of Motor Proficiency (BOT-2), cognitive function test, Cerebral Spinal Fluid (CSF)-oligosaccharides, S-oligosaccharides and specific CSF-biomarkers (CSF-tau-protein (CSF-T-tau), CSF-Glial Fibrillary Acidic protein (CSF-GFAp), CSF-NeuroFilament Light protein (CSF-NFLp)) were made only for patients included in rhLAMAN-02 and rhLAMAN-05 (n = 35).

### Biochemical assessments

#### Oligosaccharides

Lumbar puncture and blood sampling were performed for the 35 patients participating in rhLAMAN-02 and rhLAMAN-05 for measurement of CSF-oligosaccharides and serum-oligosaccharides, respectively. Oligosaccharides in CSF and serum were determined quantitatively by electrospray tandem mass spectrometry. Assays were performed at the Danish Technology Institute, Kolding, Denmark.

#### CSF-biomarkers of neurodegeneration

Lumbar puncture was performed for the 35 patients participating in rhLAMAN-02 and rhLAMAN-05 and measurement of CSF biomarkers, T-tau, NFLp and GFAp were done. Thirty four of 35 CSF-samples were analysed. The total volume of CSF was seven ml. For CSF biomarkers the CSF was frozen at -20 °C, for CSF oligosaccharides at -80 °C.

CSF T-tau was measured using an ELISA assay (INNOTEST hTau Ag, Innogenetics, Ghent, Belgium) [19]. CSF GFAP and CSF NFLp were measured using an in-house developed sandwich ELISA [20, 21]. Determination of levels of CSF biomarkers was performed at Sahlgrenska Hospital, Göteborg, Sweden.

#### Alpha-mannosidase activity

Determination of alpha-mannosidase activity was done in serum in rhLAMAN-01, and in plasma in rhLAMAN-02 and rhLAMAN-05. Both determinations were performed as described by Masson and Lundblad [22].

To make the two methods comparable, enzyme activities were reported as the percentages of the mean of the reference values.

### Clinical assessments

#### Cognitive function test

The cognitive function has been investigated in another study of ours [23]. Briefly, the Leiter International Performance Scale-Revised (Leiter R) was used [24–26]*.* The test consists of two test batteries; Visual Function and Reasoning battery and Memory and Attention battery. For each of the test batteries a measure of the overall functioning level, a total equivalent age, is obtained. All 35 patients performed the Visual Function and Reasoning battery, only 26, enrolled or screened for rhLAMAN-05, performed the Memory and Attention battery.

#### Pulmonary function test

Pulmonary function test measuring the forced vital capacity (FVC) (and percentage of predicted value, depending on age, size and sex (FVC%), was performed in accordance with American Thoracic Society (ATS)-standards and The European Respiratory Society Statements (ERS) [27].

#### Motor function assessments

6MWT was performed in accordance with ATS-standards [28]. 3MSCT is not a standardised test, but was performed according to published guidelines [29–31]. BOT-2 is a standardised and validated test measuring fine and gross motor skills of children and adolescents [32].

#### Audiometry

Unaided pure-tone audiometry at frequencies 0.25, 0.5, 1, 2 and 4 kHz was carried out in accordance with ISO 8253–1 [33].

### Statistical analysis

Statistical analyses were performed using the SPSS software. Analyses of covariance were performed to describe the genotype-phenotype correlations. The phenotype parameters were modelled by the mutation group and age as a covariate. P values of <0.05 were considered as statistically significant.

#### Sample size calculations

No formal sample size calculation was performed for this study. The sample size is from a statistical point of view a small number of persons, but it represents a compromise between the limited number of persons fulfilling the inclusion/exclusion criteria and the minimal amount of data which can support a possible genotype-phenotype correlation.

## Results

The diagnosis of alpha-mannosidosis was established in all 66 patients (24 females and 42 males), age 5 to 42 years (mean 19.4), from 57 families based on deficiency of acid alpha-mannosidase activity in leukocytes or serum.

### Mutation spectrum and functional analyses of novel missense variants

**Fig. 1:**
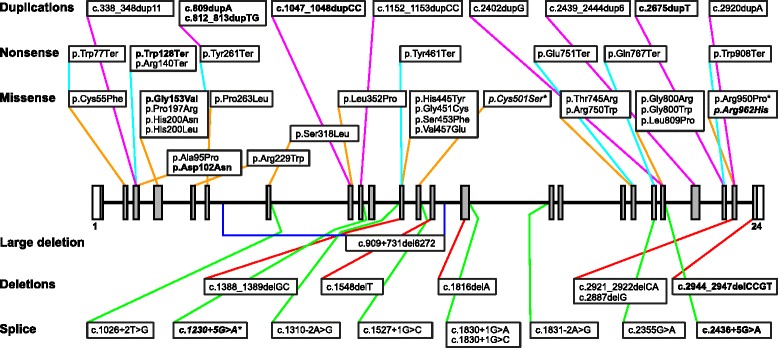
Schematic view of the localisation and type of mutations in the study cohort. Boxes represent exons (coding region in grey), lines represent introns. Mutations are labelled according to HGVS recommendations (http://www.hgvs.org/mutnomen/). Deletions, duplications and splice variants are described using the *MAN2B1* coding DNA reference sequence NM_000528.3, where position +1 corresponds to A in the first ATG translation initiation codon. Novel mutations are in bold. Variants of uncertain clinical significance are in italics. *Variant c.1230+5G>A was detected in two siblings where it was in *cis* with c.2248C>T p.Arg750Trp; variants c.1501T>A p.Cys501Ser and c.2849G>C p.Arg950Pro were in *cis* in one patient

Disease-associated *MAN2B1* mutations were identified in all patients. About half of the unrelated patients were homozygous (28 of 57). In total we detected 56 disease-associated variants, of which 11 were novel (see Tables 1 and 2 and Fig. 1). None of the novel mutations were present in dbSNP (http://www.ncbi.nlm.nih.gov/projects/SNP/) but c.2885G>A p.(Arg962His) was present in the large database of exome sequences ExAc (Exome Aggregation Consortium (www. http://exac.broadinstitute.org/)) with a frequency of 2,47e-05 (3 alleles out of 121 374). For 43 patients parental sequencing data were collected, for 7 patients only sequencing data from the mother or the father were collected and for 16 patients the parental samples were not available.

**Table 1:** Novel *MAN2B1* variants detected in this study

cDNA label	Location	Predicted effect on protein	Activity^2^	Processing^3^	Location^4^
**Nonsense mutations/duplications/deletions**					
c.383G>A	Exon 3	p.(Trp128Ter)			
c.809dupA	Exon 6	p.(Asp270GlufsTer54)			
c.812_813dupTG	Exon 6	p.(Leu272CysfsTer27)			
c.1047_1048dupCC	Exon 8	p.(His350ProfsTer15)			
c.2675dupT	Exon 22	p.(Arg893AlafsTer38)			
c.2944_2947delCCGT	Exon 24	p.(Pro982ThrfsTer50)			
**Splice site mutations**					
c.1230 + 5G>A^1^	Intron 9	p.?			
c.2436 + 5G>A	Intron 20	p.Glu786_Met812del			
**Missense mutations**					
c.304G>A	Exon 3	p.(Asp102Asn)	No	Yes	Lysosomal
c.458G>T	Exon 4	p.(Gly153Val)	No	Yes	Lysosomal
c.2885G>A^5^	Exon 22	p.(Arg962His)	Yes	Yes	Lysosomal

Mutations were labelled following the most recent Human Genome Variation Society (HGVS) guidelines (version 2.121101, www.hgvs.org/mutnomen). Mutant residues were numbered using the *MAN2B1* reference sequence NM 000528.3. The systematic names are preceded by a “c.” following the HGVS recommendations for cDNA reference numbering, with +1 as A of the initiation codon ATG. At the protein level, names are preceded by “p.”. Amino acids are listed according to the three-letter code. Protein labels are in parentheses if the variant has not been studied on RNA or protein level

^1^Located on the same allele (in *cis*) as the known pathogenic missense variant c.2248C>T p.Arg750Trp

^2^In lysates of transiently transfected COS-7 cells: No≤20 % of WT; Some = 20-30 % of WT; Yes≥30 % of WT

^3^In lysates of transiently transfected COS-7 cells and HeLa-cells

^4^ *In vivo* localisation in transiently transfected HeLa-cells

^5^Variant of uncertain clinical significance

**Table 2:** *MAN2B1* mutations and genotype/subcellular localisation subgroups for all patients

	Allele 1		Allele 2		Localisation	Genotype group^10^/subcellular localisation subgroups
Family number	Label cDNA	Label Protein	Label cDNA	Label Protein		
1A^1^	c.164G>T	p.(Cys55Phe)	c.599A>T	p.(His200Leu)	Exon 2 / Exon 4	3
1B^1^	c.164G>T	p.(Cys55Phe)	c.599A>T	p.(His200Leu)	Exon 2 / Exon 4	3
2	c.231G>A	p.(Trp77Ter)	c.2398G>C	p.(Gly800Arg)	Exon 2 / Exon 20	2*
3	c.283G>C	p.(Ala95Pro)	c.283G>C	p(.Ala95Pro)	Exon 3 / Exon 3	2
4	c.283G>C	p.(Ala95Pro)	c.283G>C	p.(Ala95Pro)	Exon 3 / Exon 3	2
5	c.304G>A	p.(Asp102Asn)	c.2885G>A	p.(Arg962His)	Exon 3 / Exon 23	3
6	c. 338_348dup11	p.(Ile117ProfsTer44)	c. 338_348dup11	p.(Ile117ProfsTer44)	Exon 3 / Exon 3	1
7	c.383G>A	p.(Trp128Ter)	c.383G>A	p.(Trp128Ter)	Exon 3 / Exon 3	1*
8	c.418C>T	p.(Arg140Ter)	c.418C>T	p.(Arg140Ter)	Exon 3 / Exon 3	1
9A^2^	c.458G>T	p.(Gly153Val)	c.[1230+5G>A;c.2248C>T]	p.[?; Arg750Trp]	Exon 4 / Intron 9	3
9B^2^	c.458G>T	p.(Gly153Va) l	c.[1230+5G>A; c.2248C>T] ^11^	p.[?; Arg750Trp]	Exon 4 / Intron 9	3
10	c.590C>G	p.(Pro197Arg)	c.2724G>A	p.(Trp908Ter)	Exon 4 / Exon 22	2*
11	c.598C>A	p.(His200Asn)	c.1548delT	p.(Leu518TrpfsTer5)	Exon 4 / Exon 13	3*
12	c.685C>T	p.(Arg229Trp)	c.2439_2444dup6	p.(His814_Arg815dup)	Exon 5 / Exon 21	3*
13	c.783C>A	p.(Tyr261Ter)	c.783C>A	p.(Tyr261Ter)	Exon 6 / Exon 6	1
14	c.788C>T	p.Pro263Leu	c.2355G>A	p.(Arg757MetfsTer6)	Exon 6 / Exon 19	3
15	c.809dupA	p.(Asp270GlufsTer54)	c.2675dupT	p.(Arg893AlafsTer38)	Exon 6 / Exon 22	1
16	c.812_813dupTG	p.(Leu272CysfsTer27)	c.812_813dupTG	p.(Leu272CysfsTer27)	Exon 6 / Exon 6	1
17	c.909+731del6272	p.Gly304del245	c.953C>T	p.Ser318Leu	Intron 6-13 / Exon 7	3
18	c.1026+2 T>G	p.[Val339_Ala341del; p.Val339_Gln342del]	c.1830+1G>C	p.Val549_Glu610del	Intron 7 / Intron 14	2
19	c.1047_1048dupCC	p.(His350ProfsTer15)	c.2248C>T	p.Arg750Trp	Exon 8 / Exon 18	2
20A^3^	c.1055 T>C	p.(Leu352Pro)	c.1055 T>C	p.(Leu352Pro)	Exon 8 / Exon 8	2
20B^3^	c.1055 T>C	p.(Leu352Pro)	c.1055 T>C	p.(Leu352Pro)	Exon 8 / Exon 8	2
21	c.1152_1153dupCC	p.(His385ProfsTer93)	c.1831-2A>G	p.His611GlyfsTer3	Exon 9 / Intron 14	1
22	c.1152_1153dupCC	p.(His385ProfsTer93)	c.1152_1153dupCC	p.His385ProfsTer93	Exon 9 / Exon 9	1
23	c.1310-2A>G	p.(?)	c.2248C>T	p.Arg750Trp	Intron 10 / Exon 18	2
24	c.1333C>T	p.(His445Tyr)	c.1333C>T	p.(His445Tyr)	Exon 11 / Exon 11	2
25	c.1351G>T	p.(Gly451Cys)	c.[1501 T>A; 2849G>C] ^11^	p.([Cys501Ser; Arg950Pro])	Exon 11 / Exon 12	3
26A^4^	c.1358C>T	p.(Ser453Phe)	c.1358C>T	p.(Ser453Phe)	Exon 11 / Exon 11	2
26B^4^	c.1358C>T	p.(Ser453Phe)	c.1358C>T	p.(Ser453Phe)	Exon 11 / Exon 11	2
27A^5^	c.1370 T>A	p.(Val457Glu)	c.2248C>T	p.Arg750Trp	Exon 11 / Exon 18	3*
27B^5^	c.1370 T>A	p.(Val457Glu)	c.2248C>T	p.Arg750Trp	Exon 11 / Exon 18	3*
28A^6^	c.1383C>A	p.(Tyr461Ter)	c.2402dupG	p.(Ser802GlnfsTer129)	Exon 11 / Exon 20	1*
28B^6^	c.1383C>A	p.(Tyr461Ter)	c.2402dupG	p.(Ser802GlnfsTer129)	Exon 11 / Exon 20	1*
29	c.1388_1389delGC	p.(Arg463ProfsTer53)	c.2426 T>C	p.Leu809Pro	Exon 11 / Exon 20	2*
30	c.1527+1G>C	p.(?)	c.1527+1G>C	p.(?)	Intron 12 / Intron 12	1*
31A^7^	c.1816delA	p.Thr606ProfsTer18	c.1830+1G>C	p.Val549_Glu610del	Exon 14 / Intron 14	1
31B^7^	c.1816delA	p.Thr606ProfsTer18	c.1830+1G>C	p.Val549_Glu610del	Exon 14 / Intron 14	1
32	c.1830+1G>A	p.(?)	c.2248C>T	p.Arg750Trp	Intron 14 / Exon 18	2
33	c.1830+1G>C	p.Val549_Glu610del	c.1830+1G>C	p.Val549_Glu610del	Intron 14 / Intron 14	1
34	c.1830+1G>C	p.Val549_Glu610del	c.2248C>T	p.Arg750Trp	Intron 14 / Exon 18	2
35	c.1830+1G>C	p.Val549_Glu610del	c.2426 T>C	p.Leu809Pro	Intron 14 / Exon 20	2
36	c.1830+1G>C	p.Val549_Glu610del	c.2248C>T	p.Arg750Trp	Intron 14 / Exon 18	2
37A^8^	c.1831-2A>G	p.His611GlyfsTer3	c.1831-2A>G	p.His611GlyfsTer3	Intron 14 / Intron 14	1
37B^8^	c.1831-2A>G	p.His611GlyfsTer3	c.1831-2A>G	p.His611GlyfsTer3	Intron 14 / Intron 14	1
38	c.2234C>G	p.(Thr745Arg)	c.2234C>G	p.Thr745Arg	Exon 18 / Exon 18	3
39A^9^	c.2248C>T	p.Arg750Trp	c.2248C>T	p.Arg750Trp	Exon 18 / Exon 18	2*
39B^9^	c.2248C>T	p.Arg750Trp	c.2248C>T	p.Arg750Trp	Exon 18 / Exon 18	2*
40	c.2248C>T	p.Arg750Trp	c.2248C>T	p.Arg750Trp	Exon 18 / Exon 18	2*
41	c.2248C>T	p.Arg750Trp	c.2248C>T	p.Arg750Trp	Exon 18 / Exon 18	2*
42	c.2248C>T	p.Arg750Trp	c.2248C>T	p.Arg750Trp	Exon 18 / Exon 18	2*
43	c.2248C>T	p.Arg750Trp	c.2248C>T	p.Arg750Trp	Exon 18 / Exon 18	2*
44	c.2248C>T	p.Arg750Trp	c.2299C>T	p.Gln767Ter	Exon 18 / Exon 19	2*
45	c.2248C>T	p.Arg750Trp	c.2248C>T	p.Arg750Trp	Exon 18 / Exon 18	2
46	c.2248C>T	p.Arg750Trp	c.2248C>T	p.Arg750Trp	Exon 18 / Exon 18	2*
47	c.2248C>T	p.Arg750Trp	c.2426 T>C	p.Leu809Pro	Exon 18 / Exon 20	2*
48	c.2248C>T	p.Arg750Trp	c.2248C>T	p.Arg750Trp	Exon 18 / Exon 18	2
49	c.2248C>T	p.Arg750Trp	c.2426 T>C	p.Leu809Pro	Exon 18 / Exon 20	2
50	c.2248C>T	p.Arg750Trp	c.2251G>T	p.(Glu751Ter)	Exon 18 / Exon 20	2*
51	c.2248C>T	p.Arg750Trp	c.2248C>T	p.Arg750Trp	Exon 18 / Exon 18	2
52	c.2355G>A	p.Arg757MetfsTer6	c.2355G>A	p.(Arg757MetfsTer6)	Exon 19 / Exon 19	1
53	c.2398G>T	p.(Gly800Trp)	c.2944_2947delCCGT	p.(Pro982ThrfsTer50)	Exon 20 / Exon 24	2
54	c.2436+5G>A	p.Glu786_Met812del	c.2887delG	p.(Glu963ArgfsTer70)	Intron 20 / Exon 23	1
55	c.2724G>A	p.(Trp908Ter)	c.2724G>A	p.(Trp908Ter)	Exon 22 / Exon 22	1
56	c.2920dupA	p.(Thr974AsnfsTer81)	c.2920dupA	p.(Thr974AsnfsTer81)	Exon 23 / Exon 23	1*
57	c.2921_2922delCA	p.(Thr974ArgfsTer80)	c.2921_2922delCA	p.(Thr974ArgfsTer80)	Exon 23 / Exon 23	1

Mutations are labelled according to HGVS recommendations (http://www.hgvs.org/mutnomen/) using the *MAN2B1* coding DNA reference sequence NM_000528.3, where position +1 corresponds to A in the first ATG translation initiation codon. Novel mutations are in bold. Protein labels are in parentheses if the variant has not been studied on RNA or protein level.

^1-9^Sibship 1-9, ^10^Subgroup 1: Two null-mutations (nonsense, frameshift, large truncations), subgroup 2: At least one missense mutation (or in-frame deletion/duplication of 1-5 amino acids) with the MAN2B1 protein localised to the endoplasmic reticulum (ER) (ie: ER/ER, ER/null), subgroup 3: At least one missense mutation (or in-frame deletion/duplication of 1-5 amino acids) with the MAN2B1 protein localised to the lysosomes (lyso) (ie: lyso/lyso, lyso/ER, lyso/null)

*Incomplete or no parental sequencing data available

Mutations were scattered all over the gene and included missense mutations (23, 3 novel), small duplications (9, 4 novel), nonsense mutations (8, 1 novel), splice site mutations (8, 1 novel), small deletions (6, 1 novel), one large deletion (intron 6-13) and one novel intronic variant (c.1230+5G>A).

Based on cDNA analyses in blood, the variant c.2436+5G>A in intron 20 caused skipping of exon 20 and deleted 81 nt from the transcript (r.2356_2436del81, p.Glu786_Met812del27). The effect of c.1230+5G>A in intron 9 was not clear from the cDNA analysis and requires further analyses. It was thus classified as a variant of unknown clinical significance (VUS). However, the variant was detected in two Spanish sibs, and in this family it was on the same allele (*in cis*) as the known disease-causing variant c.2248C>T (p.Arg750Trp). All nonsense mutations, frame-shifting small deletions/duplications, large truncations caused by splice site mutations and the large deletion were considered null-alleles.

**Fig. 2:**
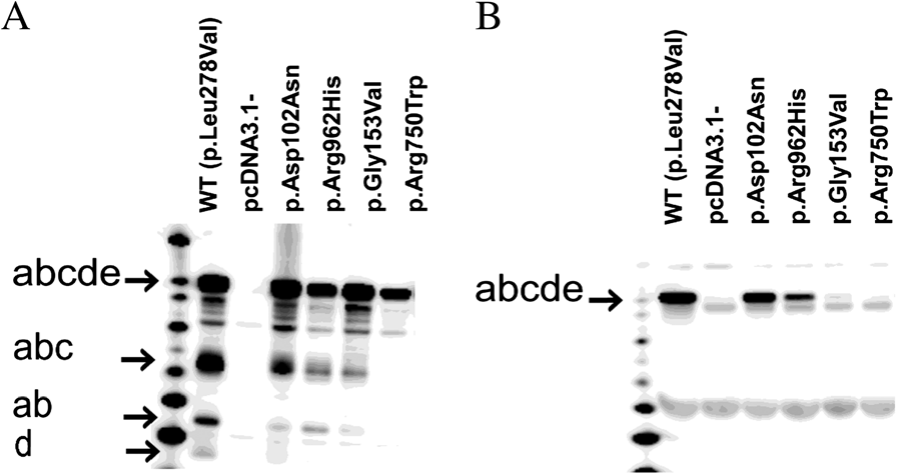
Western blot showing the intracellular processing and secretion of the novel MAN2B1 missense variants in transfected COS-7 cells. The relative intensity of the different peptides is different for the wild-type enzyme and the missense variants. **a.** Overexpressed and transported MAN2B1 proteins are also secreted into the cell media in the full-length form. **b.** The WT was included as a positive control of MAN2B1 processing/secretion, pcDNA3.1 was included as a negative control of MAN2B1 expression (cells transfected with an empty vector) and MAN2B1 p.Arg750Trp was included as a negative control of MAN2B1 processing/secretion (accumulates in the ER)

**Fig. 3:**
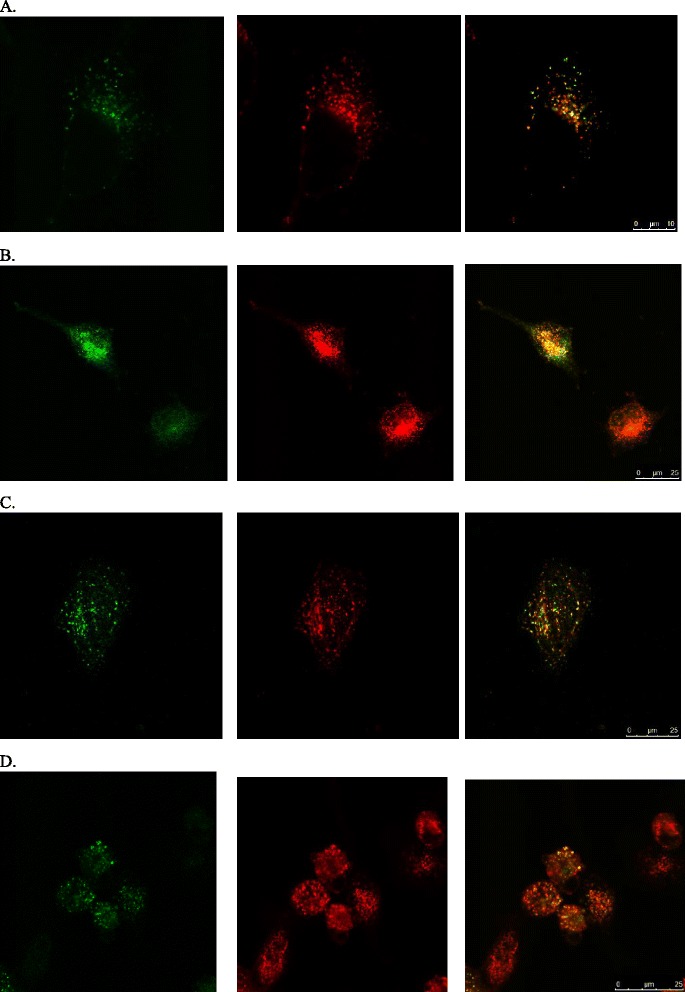
Confocal fluorescent microscopy images showing the intracellular localisation of the novel MAN2B1 missense variants in transfected HeLa-cells. The first column of images shows methanol-fixed transfected HeLa-cells stained for MAN2B1 (green), the second column shows the same cells stained for the lysosomal marker LAMP1 (red) and the third column shows merged images with colocalized MAN2B1 and LAMP1 (yellow). **a**: MAN2B1 p.Asp102Asn; **b**: MAN2B1 p.Gly153Val; **c**: MAN2B1 p.Arg962His; **d**: WT. The WT was included as a positive control of lysosomal localization

In total, 23 missense mutations were detected in our patient cohort. Of these, 3 were novel; c.304G>A (p.Asp102Asn), c.458G>T (p.Gly153Val) and c.2885G>A (p.Arg962His). Based on transfection experiments in cell culture, MAN2B1 p.Asp102Asn and p.Gly153Val were found to be inactive, processed correctly, secreted into the medium and localised to the lysosomes (Figs. 2 and 3). MAN2B1 p.Arg962His showed considerable residual activity indistinguishable from the activity observed for common, non-pathogenic variants [7] and in some experiments resembling the level of the WT, and was processed correctly and localised to the lysosomes. However, the intensity of the bands representing the processed peptides (as seen on western blot) was weaker for the variant as compared to the wild-type MAN2B1 (Figs. 2 and 3), and it was consequently classified as a VUS.

### Genotype-phenotype correlations

Although all the patients included in this study were classified as having the attenuated – type II alpha-mannosidosis, their clinical phenotypes demonstrated a large degree of variability. On the basis of the mutation analysis, and the predicted effect of the mutations, the patients were divided into the genotype/subcellular localisation subgroups. The distribution of patients and ages in the three genotype/subcellular localisation subgroups are shown in Table 3.

**Table 3:** The distribution of patients in the three subgroups

Genotype/subcellular localisation subgroups	N	Age +/-SD, Mean (years)	Age Median (years)	Age Minimum (years)	Age Maximum (years)
Subgroup 1	21	22.9 ± 11.6	23.0	5.7	42.1
Two null-mutations (nonsense, frameshift, large truncation)					
Subgroup 2	32	17.9 ± 7.6	17.9	6.0	35.0
Missense/in-frame dup/del localised to the ER					
Subgroup 3	13	17.4 ± 7.8	17.3	5.4	29.0
Missense/in-frame dup/del localised to the lysosomes					
Total	66	19.4 ± 9.3	17.3	5.4	42.1

**Fig. 4:**
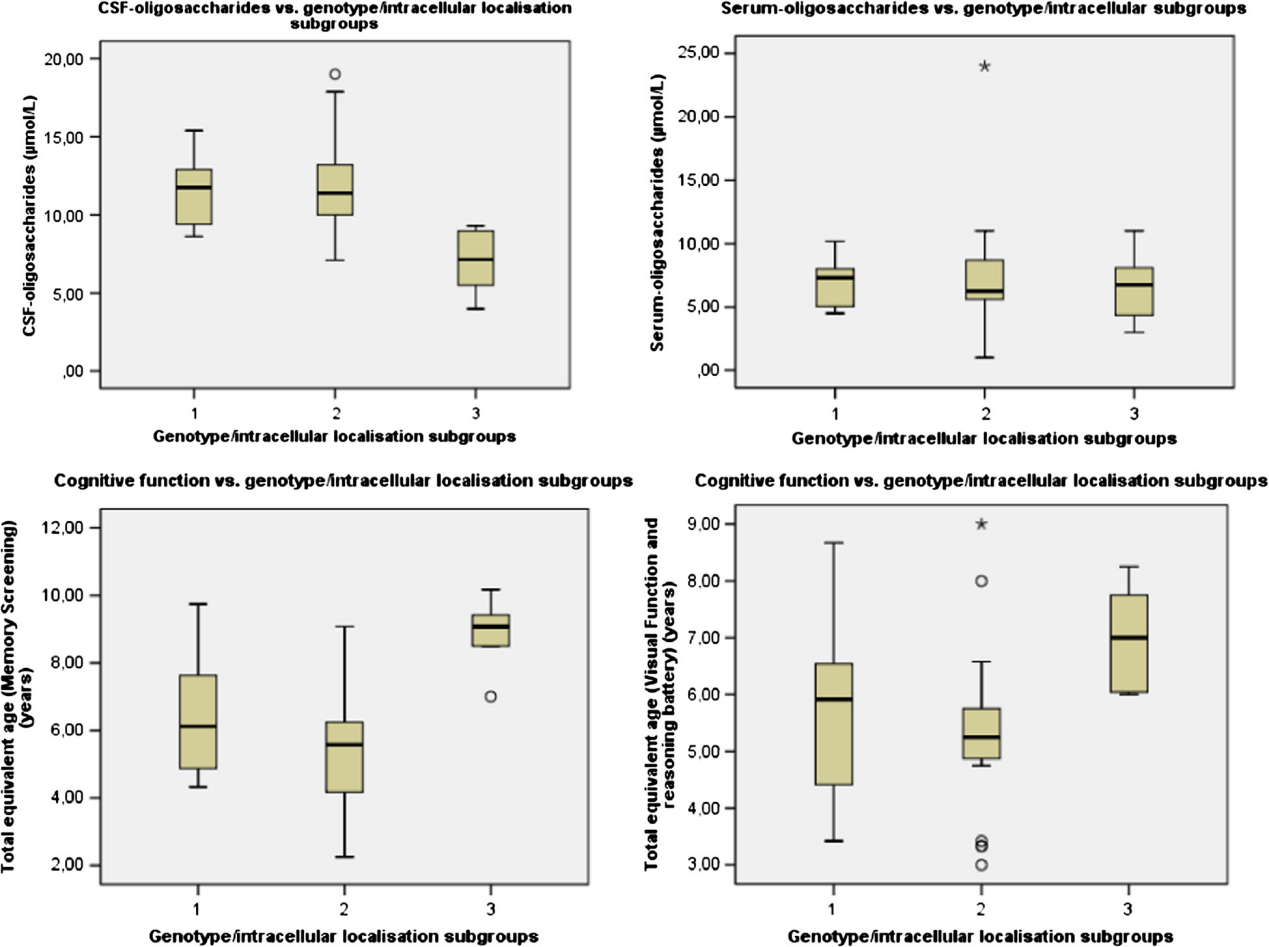
The correlation between the three genotype/subcellular localisation subgroups and CNS related clinical and biochemical data. The correlation between the three genotype/subcellular localisation subgroups and serum-oligosaccharides, CSF-oligosaccharides, total equivalent age for Visual Function and Reasoning battery and Total equivalent age for Memory and Attention battery. (CSF-oligosaccharides: H_0_ 3 = 2: p = 0.001, H_0_ 3 = 1: p = 0.011, H_0_ 2 = 1: p = 1.000, Serum-oligosaccharides: p = 0.76 (age p = 0.86) (Because of the non-significant results, pairwise comparisons are not reported), Total equivalent age for Visual Function and Reasoning battery (H_0_ 3 = 2: p = 0.02, H_0_ 3 = 1: p = 0.215, H_0_ 2 = 1: p = 0.836), Total equivalent age for Memory and Attention battery (H_0_ 3 = 2: p = 0.296, H_0_ 3 = 1: p = 0.003, H_0_ 2 = 1: p = 0.042)

**Fig. 5:**
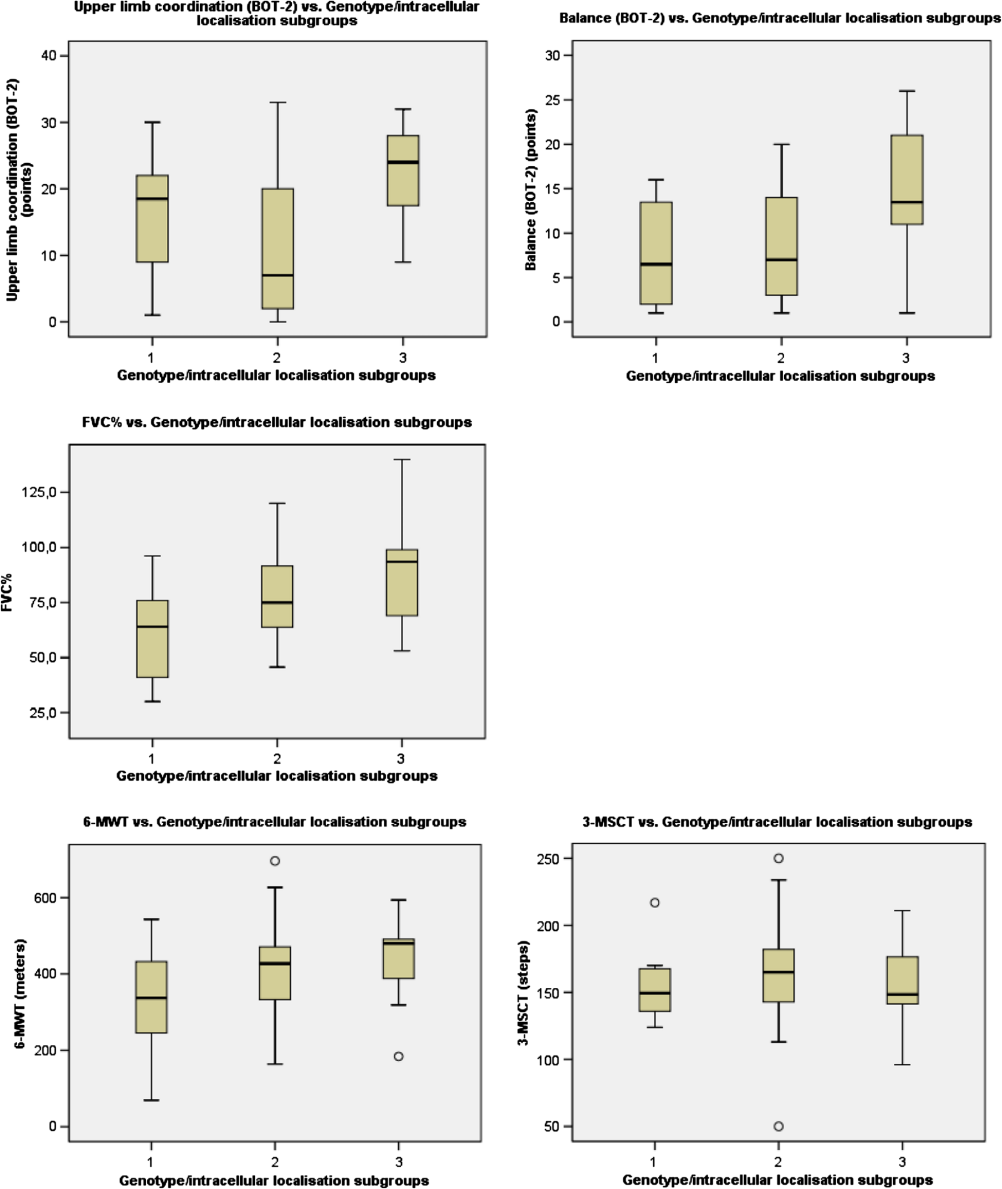
Correlation between the three genotype/subcellular localisation subgroups and the motor function test and FVC%. BOT-2 subtest: Balance: H_0_ 3 = 2: p = 0.033, H_0_ 3 = 1: p = 0.06, H_0_ 2 = 1: p = 1.000, BOT-2 subtest: Upper limb and coordination: H_0_ 3 = 2: p = 0.047, H_0_ 3 = 1: p = 0.713, H_0_ 2 = 1: p = 0.773, FVC%: H_0_ 3 = 2: p = 0.296, H_0_ 3 = 1: p = 0.003, H_0_ 2 = 1: p:0.042. 6-MWT (six-minutes-walk-test): p = 0.102 (age: p = 0.01), 3-MSCT (three-minutes-stair-climb-test): p=0.82 (age p=0.60). Because of the non-significant results, pairwise comparisons are not reported

The correlation between the three genotype/subcellular localisation subgroups and CNS related clinical and biochemical data are shown in Fig. 4, the correlation between the three genotype/subcellular localisation subgroups and the motor function test and FVC% are shown in Fig. 5.

Beside the significant correlations demonstrated in Table 4, correlations between the genotype/subcellular localisation subgroups, including pairwise comparison, and S-oligosaccharides; CSF-biomarkers; alpha-mannosidase activity (%); 6-MWT; 3-MSCT and audiometry were performed. No significant correlations were found (S-oligosaccharides: p=0.76 (age p=0.86), CSF-GFAp: p=0.11 (age p=0.94), CSF-T-tau: p=0.12 (age p=0.006), CSF-NFLp: p=0.46 (age p=0.18), alpha-mannosidase activity (%): p=0.19 (age p=0.10), 6-MWT: p=0.102 (age p=0.01): 3-MSCT: p=0.82 (age p=0.60), Bone conducted hearing loss: p=0.90 (age p=0.001): Air conducted hearing loss: p=0.37 (age p=0.26). Because of the non-significant results, pairwise comparisons are not reported.

**Table 4:** Correlations between the three genotype/subcellular localisation subgroups and biochemical and clinical test results

Analysis/clinical test	N	Subgr. 1, mean	Subgr. 2, mean	Subgr. 3, mean	Min.	Max.	Total, mean	SD	p-value Pairwise comparison*
CSF-oligo (μmol/L)	35/35	11.5 ± 2.3	11.9 ± 3.2	7.1 ± 2.0	4.0	19	10.7	3.4	H_0_ 3 = 2: p = 0.001, H_0_ 3 = 1: p = 0.011
									H_0_ 2 = 1: p = 1.000
Cognitive test (VR battery) (years)	35/35	5.8 ± 1.7	5.3 ± 1.5	7.0 ± 0.9	3.0	9.0	5.8	1.6	H_0_ 3 = 2: p = 0.049, H_0_ 3 = 1: p = 0.344,
									H_0_ 2 = 1: p = 1.000
Cognitive test (Memory Screening) (y)	26/26	6.9 ± 1.9	5.4 ± 2.0	8.8 ± 1.2	2.3	10.2	6.5	2.3	H_0_ 3 = 2: p = 0.02, H_0_ 3 = 1: p = 0.215,
									H_0_ 2 = 1: p = 0.836
FVC% (%)	54/66*	62.3 ± 20.7	77.5 ± 19 %	90.7 ± 22.7	30	140	75.2	22.7	H_0_ 3 = 2: p = 0.296, H_0_ 3 = 1: p = 0.003,
									H_0_ 2 = 1: p = 0.042
BOT-2 (Coor.) (points)	35/35	16.3 ± 10.1	11.3 ± 10.8	22.5 ± 7.6	1	33	15	10.8	H_0_ 3 = 2:p = 0.047, H_0_ 3 = 1: p = 0.713
									H_0_ 2 = 1: p = 0.773
BOT-2 (Balance) (points)	35/35	7.6 ± 6.2	8.4 ± 6.1	14.8 ± 7.9	1	26	9.7	7.0	H_0_ 3 = 2: p = 0.033, H_0_ 3 = 1: p = 0.06,
									H_0_ 2 = 1:p = 1.00

Significant correlations between the three genotype/subcellular localisation subgroups and CSF-oligosaccharides (CSF-oligo), Cognitive test (VR battery) (=Total equivalent age for the Visual function and reasoning battery), Cognitive test (Memory Screening) (Total equivalent age for the Memory Screening), Forced Vital Capacity (FVC%), BOT-2 (Upper limb coordination) (Coor.) and BOT-2 (Balance)

N = number, min. = minimum, Max. = Maximum, SD = standard deviation, * H_0_ 3 = 2/ H_0_ 3 = 1/ H_0_ 2 = 1: Refers to the null hypothesis (H_0_) for the comparison of subgroup 3 versus subgroup 2 / subgroup 3 versus subgroup 1 / subgroup 2 versus subgroup 1. p < 0.05 rejects the H_0_

*54/66 were able to perform a spirometry

## Discussion

Two main findings have been obtained in this first systematic study of the potential relationship between genotype and phenotype in alpha-mannosidosis.

Firstly, by analysing 66 patients from 57 families, 56 disease-associated mutations were identified. Complete *MAN2B1* genotypes were established for all patients included.

Forty five mutations were described previously and 11 were novel. In accordance with previous findings, the mutations were scattered all over the *MAN2B1* gene and included most types of mutations. The most frequent missense mutation, c.2248C>T (p.Arg750Trp), was detected in 20 unrelated patients from 9 countries and accounted in this study for 25.4 % of the disease alleles in unrelated patients, which is in accordance with previous reports [11, 16].

Secondly, we found that genotypes, allowing mutant MAN2B1 protein to enter the lysosomes, correlated positively with several clinical and biochemical parameters.

Based on the predicted severity of the genotypes and subcellular localisation of the mutant MAN2B1 protein, we classified our patients into three subgroups. We hypothesised that patients from subgroups 1 and 2, either with two null-mutations or production of incorrectly folded MAN2B1 proteins were expected to have the most severe phenotype. The patients in subgroup 3, with at least one variant that allows localisation of the mutant MAN2B1 protein to the lysosomes, were hypothesised to show the mildest clinical presentation. They were expected to produce MAN2B1 proteins which fold sufficiently to reach the lysosomes and which potentially could confer some residual MAN2B1 activity.

As hypothesised, patients from subgroup 3, harbouring at least one variant that allows localisation of the mutant MAN2B1 protein to the lysosomes, performed significantly better and had less abnormal results in some of the clinical tests and biochemical analyses, compared to patients in subgroup 2 and/or subgroup 1. Thus, in patients from subgroup 3, we found a significantly higher total equivalent age in the two batteries employed in the cognitive test, a higher point score in two BOT-2 subtests (Upper limb coordination and Balance), a higher FVC% and lower concentrations of CSF-oligosaccharides compared to the other groups.

Our findings indicate a correlation between genotype/intercellular localisation and the impact on CNS, pulmonary function and motor function. The genotype-phenotype correlation concerning the CNS function, are supported by a significant negative correlation between level of CSF-oligosaccharides and level of cognitive function in the same cohort; the higher level of CSF-oligosaccharides, and the lower a total equivalent age in the Memory Screening (p = 0.039 (age: p = 0.136) [23].

The significantly better performance of subgroup 3 patients in BOT-2 subtests (Upper limb coordination and Balance), may indicate a genotype-phenotype correlation concerning balance and muscular coordination. Disabilities in muscular coordination and balance are well known and important clinical features [1, 34], which makes a genotype-phenotype correlation concerning these skills particularly interesting. A tendency to a genotype-phenotype correlation concerning the 6-MWT was also found, which might indicate a relationship between the different genotype/subcellular localisation subgroups and motor function in general.

Due to intellectual disabilities, correctly performed spirometry has been a challenge for some alpha-mannosidosis patients. Thus, the values given at spirometry may be inaccurate for some of the patients [17, 18] and the relationship between the FVC% and the different subgroups may be uncertain.

No significant correlation between genotype/subcellular localisation subgroups and lysosomal alpha-mannosidase residual activity was found, though previous studies have suggested that severity of clinical expression is related to the degree of reduced enzyme activity in human alpha-mannosidosis patients [35]. However, other studies are in accordance with our results [7, 36]. It has been suggested that the missing correlation might be due to alpha-mannosidases from other cellular compartments that confer residual activity at low pH. This may be misinterpreted as residual lysosomal alpha-mannosidase activity [16]. Moreover, mutant MAN2B1 proteins, mislocalised to non-lysosomal compartments (and thus incapable of lysosomal glycoprotein degradation), may show some activity when released into serum from leukocytes [7].

Measuring residual lysosomal alpha-mannosidase activity is difficult. The values for alpha-mannosidase activities are low in all patients; hence, even minor potential sources of error may influence the results significantly and obscure a genotype/phenotype correlation. Development of more accurate methods is necessary for final conclusions of a genotype-phenotype correlation concerning residual lysosomal alpha-mannosidase activity.

Previously, genotype-phenotype correlations in alpha-mannosidosis have been investigated in two studies [11, 16]. Our findings are not in line with those of Berg *et al.* [16] and Riise Stensland *et al.* [11], who found no apparent genotype-phenotype correlation when investigating the relationship between different types of mutations and the clinical subtype classification in 23 and 130 patients, respectively. These studies classified the patients into three phenotypic subgroups, type 1, type 2 and type 3, according to the severity of manifestations as suggested by Malm and Nilssen [34, 37]. Both studies were conducted at a time before proper clinical end-points were established. A challenge using these three phenotypic subgroups is that most of the patients diagnosed with alpha-mannosidosis are intermediate type 2 patients. In the study by Riise Stensland *et al.* [11], 106 of 130 patients were classified as type 2, and in Berg *et al.* [16], the clinical data were not collected in a standardised format, but 22 of 23 patients were described with moderate clinical manifestations.

Our cohort has not been classified into the above three phenotypic subtypes, since the disease manifestations display a continuum of clinical severity, as documented from our large cohort of alpha-mannosidosis patients and newer literature [1]. Instead, individual results from clinical and biochemical tests were used to investigate the relationship with the three genotype/subcellular localisation subgroups, making a more detailed and precise evaluation possible.

Our data have been collected in a standardised manner. For rhLAMAN-02 and rhLAMAN-05 the same doctor/nurse/ physiotherapist *etc.* assisted the patients when performing the tests. For the two previous studies [11, 16], clinical information for most of the patients was collected by the referring physicians, which could possibly introduce dissimilarity in the reporting.

Our findings demonstrated that genotypes allowing mutant MAN2B1 protein to enter the lysosomes, correlated positively with several clinical and biochemical parameters, though further investigation of a larger cohort will be necessary before such correlation can be used in clinical practice for postnatal or prenatal prognostics. Despite our findings challenges still remain.

One challenge is a phenotypic variation within sibships with identical *MAN2B1* mutations [12–15, 38]. Riise Stensland *et al.* [11] described 16 sibships with alpha-mannosidosis classified in phenotypic subgroups 1-3, and one was discordant. Among our patients participating in rhLAMAN-02 or rhLAMAN-05, one of three sibships presented with different degrees of disease involvement, especially with regard to the cognitive function, though both patients in this sibship belonged to the attenuated type 2. The most affected sibling in this sibship was treated twice with protracted mechanical ventilation during the first 1.5 years of life, due to Respiratory Syncytial Virus and bacterial pneumonia, which may have influenced the severity of the phenotype, including the cognitive function. Based on our and Riise Stensland *et al.*’s findings, the phenotypic variation between sibs might not be as frequent as previously reported, and may be explained by environmental and/or epigenetic factors.

Another challenge is the fact that some patients clinically characterised as less severely or moderately affected are homozygous for null mutations [16]. A possible explanation to this phenomenon is that other mannosidases than lysosomal alpha-mannosidase, may contribute to salvage pathways in the degradation of glycoproteins. Evidence for a role of cytosolic alpha-mannosidase in the subcellular degradation of oligosaccharides was provided by Grard *et al.* [39, 40]. Hence, it is possible that allelic heterogeneity among extra-lysosomal alpha-mannosidases could influence the phenotypic expression of alpha-mannosidosis.

In view of the above considerations, we believe that the genotype is fundamental in determining the severity of alpha-mannosidosis, but other factors, both genetic, epigenetic and environmental, may contribute to the observed clinical variation. As examples, occurrence of severe infections (which is common in alpha-mannosidosis), differences in education, motor and cognitive stimulation may affect the phenotype.

## Conclusion

In conclusion, this study is the first to investigate the genotype-phenotype correlation in alpha-mannosidosis by classifying the cohort into three genotypic/subcellular localisation subgroups and investigating the correlation between the subgroups and the individual results of clinical and biochemical tests. Our data indicate a genotype-phenotype relationship between the genotype/subcellular localisation subgroups and the cognitive function, the BOT-2 motoric test, FVC% and the storage of oligosaccharides in CSF. Patients from subgroup 3, with at least one mutation that allows localisation of the mutant MAN2B1 protein to the lysosomes, performed significantly better and had less abnormal results in some of the clinical tests and biochemical analyses, compared to patients in subgroup 2 and/or subgroup 1.

Further investigation of a larger cohort will be necessary before such correlation can be used in clinical practice for postnatal or prenatal prognostics.

## Details of ethics approvals

This study includes baseline data from rhLAMAN-01, rhLAMAN-02 and rhLAMAN-05. All three studies were approved by the local Ethics Committee and additionally rhLAMAN-02 and rhLAMAN-05 were approved by the Danish Medicines Agency.

EudraCT numbers: 2010-022084-36 (NCT00498420) and 2012-000979-17 (NCT01681953).

## Abbreviations

3MSCT: Three-minute Stair Climb Test
6MWT: Six-minute Walk Test
ATS: American thoracic society
BOT2: Bruininks-Oseretsky test of Motor Proficiency
CNS: Central Nervous System
CSF: Cerebrospinal Fluid
ER: Endoplasmic reticulum
ERT: Enzyme Replacement Therapy
FVC: Forced Vital Capacity
GCP: Good Clinical Practice
GFAp: Glial Fibrillary Acidic protein
Leiter R: Leiter International Performance Scale-Revised
NFLp: Neurofilament Protein
PFT: Pulmonary Function Testing
PTA: Pure Tone Audiometry
T-tau: Tau Protein
VUS: Variant of unknown clinical significance
WT: Wild-type
